# Evaluation of pulmonary arterial stiffness in post mild COVID-19 patients: a pilot prospective study

**DOI:** 10.1186/s44348-024-00032-3

**Published:** 2024-08-28

**Authors:** Yetkin Korkmaz, Tufan Çınar, Faysal Şaylık, Tayyar Akbulut, Murat Selçuk, Mustafa Oğuz, Mert Ilker Hayıroğlu, İbrahim Halil Tanboğa

**Affiliations:** 1 Department of Cardiology, Sultan II. Abdulhamid Han Training and Research Hospital, Istanbul, Türkiye; 2 Department of Cardiology, Van Training and Research Hospital, Van, Türkiye; 3 Department of Cardiology, Dr. Siyami Ersek Training and Research Hospital, Istanbul, Türkiye; 4 Department of Cardiology and Biostatistics, Nisantasi University Medical School, Istanbul, Türkiye

**Keywords:** Pulmonary artery stiffness, Pulmonary Arterial Hypertension, COVID-19

## Abstract

**Background:**

Our primary goal was to utilize pulmonary arterial stiffness (PAS) to demonstrate the early alterations in the pulmonary vascular area in individuals with prior COVID-19 illness who had not undergone hospitalization.

**Methods:**

In total, 201 patients with prior COVID-19 infection without hospitalization and 195 healthy, age- and sex-matched individuals without a history of COVID-19 disease were included in this prospective analysis. The PAS value for each patient was calculated by dividing the mean peak pulmonary flow velocity by the pulmonary flow acceleration time.

**Results:**

The measured PAS was 10.2 ± 4.11 Hz/msec in post–COVID-19 participants and 8.56 ± 1.47 Hz/msec in healthy subjects (*P* < 0.001). Moreover, pulmonary artery acceleration time was significantly lower in patients with a prior history of COVID-19. Multivariable logistic regression analysis revealed that PAS was significantly connected to a prior COVID-19 illness (odds ratio, 1.267; 95% confidence interval, 1.142–1.434; *P* < 0.001). The optimal cutoff point for detecting a prior COVID-19 disease for PAS was 10.1 (sensitivity, 70.2%; specificity, 87.7%).

**Conclusions:**

This might be the first investigation to reveal that patients with a history of COVID-19 had higher PAS values compared to those without COVID-19. The results of the investigation may indicate the need of regular follow up of COVID-19 patients for the development of pulmonary arterial hypertension, especially during the post–COVID-19 interval.

## Background

Pulmonary arterial hypertension (PAH) is a challenging condition with high mortality and morbidity that early diagnosis might be lifesaving. Pulmonary arterial stiffness (PAS) is established as an echocardiographic parameter to examine the vasculature and physiomechanics of the pulmonary arterial (PA) system. The assessment PAS, in particular during the early stages of PAH, may provide a reliable risk prediction for the development of severe PAH and concomitant mortality [1, 2]. Earlier studies found a significant relationship between the PAS and the measurements obtained from right cardiac catheterizations [3, 4]. In addition, increased PAS in children with congenital heart disease has been shown to accurately predict progression to advanced PAH, even in patients with low pulmonary vascular resistance [5]. This suggests that intrinsic PAS potentially function as a separate risk indicator to enhance prognoses of disease development and survival.

In the end of 2019, a cluster of cases of pneumonia were discovered in the city of Wuhan, and it was determined that the SARS-CoV-2, a new variant of the coronavirus, was responsible for this illnesses [6]. Even while the majority of SARS-CoV-2 infections only produce relatively minor symptoms such as fever, coughing, and the upper respiratory tract symptoms, it still has the risk of leading to complications like as pneumonia and acute respiratory distress syndrome. It is generally agreed upon that treating the cardiovascular and pulmonary consequences of COVID-19 is necessary in order to reduce the severity and the death rate associated with it. On the other hand, neither the short-term nor the long-term consequences of a mild infection in the pulmonary vascular bed have been fully investigated. Additionally, individuals who have recovered from different coronavirus infections, such as Middle Eastern respiratory syndrome (MERS) and severe acute respiratory syndrome (SARS), have been demonstrated to have a gradual decline in their respiratory functioning [7–10]. In this study, we investigated whether there was a significant change in PAS in patients diagnosed with COVID-19 who survived the condition without requiring hospitalization.

## Methods

### Study participants

In this prospective investigation, individuals who applied to the cardiology clinic between July 2022 and August 2022 for regular control and had a history of COVID-19 illness in the past 3 to 6 months without a need for hospitalization were included. There were studies in the literature stating COVID-19 symptoms could last up to 12 weeks and the period after 12 weeks could be defined as the post–COVID-19 [11]. Again, in the literature, there were studies conducted in up to the 6th month in COVID-19 patients [11]. We did not keep this period longer in order to exclude possible alternative diseases that could affect a sensitive evaluation such as PAS. To confirm COVID-19 infection in all patients, real-time reverse transcriptase polymerase chain reaction analysis on nasopharyngeal swab was used. None of the patients exhibited signs of an active COVID-19 illness at the time of examination. In total, 195 healthy volunteers with similar age, sex, and body mass index compromised the control group. We made sure that the healthy volunteers we took as the control group did not have a clinical history that could be considered as COVID-19 symptoms in the last 6 months. Cardiovascular, pulmonary, systemic, and viral diseases associated with PAH and those with a history of drug use that may be associated with PAH were excluded. Furthermore, we excluded individuals with chronic conditions such as essential hypertension, diabetes mellitus, chronic kidney failure, and rheumatic diseases. Age, sex, and body mass index were all reported as baseline clinical information for all patients.

### Transthoracic echocardiographic evaluation

Transthoracic echocardiographic examination was performed by two skilled echocardiographers who were not given access to the patients' medical records. All patients got comprehensive transthoracic echocardiographic evaluations as recommended by the American Society of Echocardiography. The left ventricular ejection fraction and end-systolic and end-diastolic volumes were evaluated from apical two- and four-chamber images using the standard biplane Simpson technique. Left ventricular end-diastolic diameter, posterior wall thickness, ventricular septum, and left atrial (LA) anteroposterior diameter were all measured from the parasternal long-axis view. The right ventricular myocardial performance index is a global calculation of myocardial function that reflects both systolic and diastolic function of the right ventricle (RV). In fact, RV MPI is a comparison of isovolumic time without ejection with ejection. The RV MPI is a global measure of right ventricular function as the ratio of isovolumic relaxation and contraction times to RV ejection time (ET). The sum of isovolumic times is calculated based on the difference between the time from tricuspid valve closure to opening (TVCOt) and RV ET from RV outflow tract sampling. RV MPI was calculated according to the formula “(TVCOt – RV ET) / RV ET.” RV systolic pressure was considered equal to PA systolic pressure, as none of the participants had pulmonary stenosis or RV outflow tract obstruction. The simplified Bernoulli equation was used to obtain the RV systolic pressure.

### PAS measurement

**Fig. 1:**
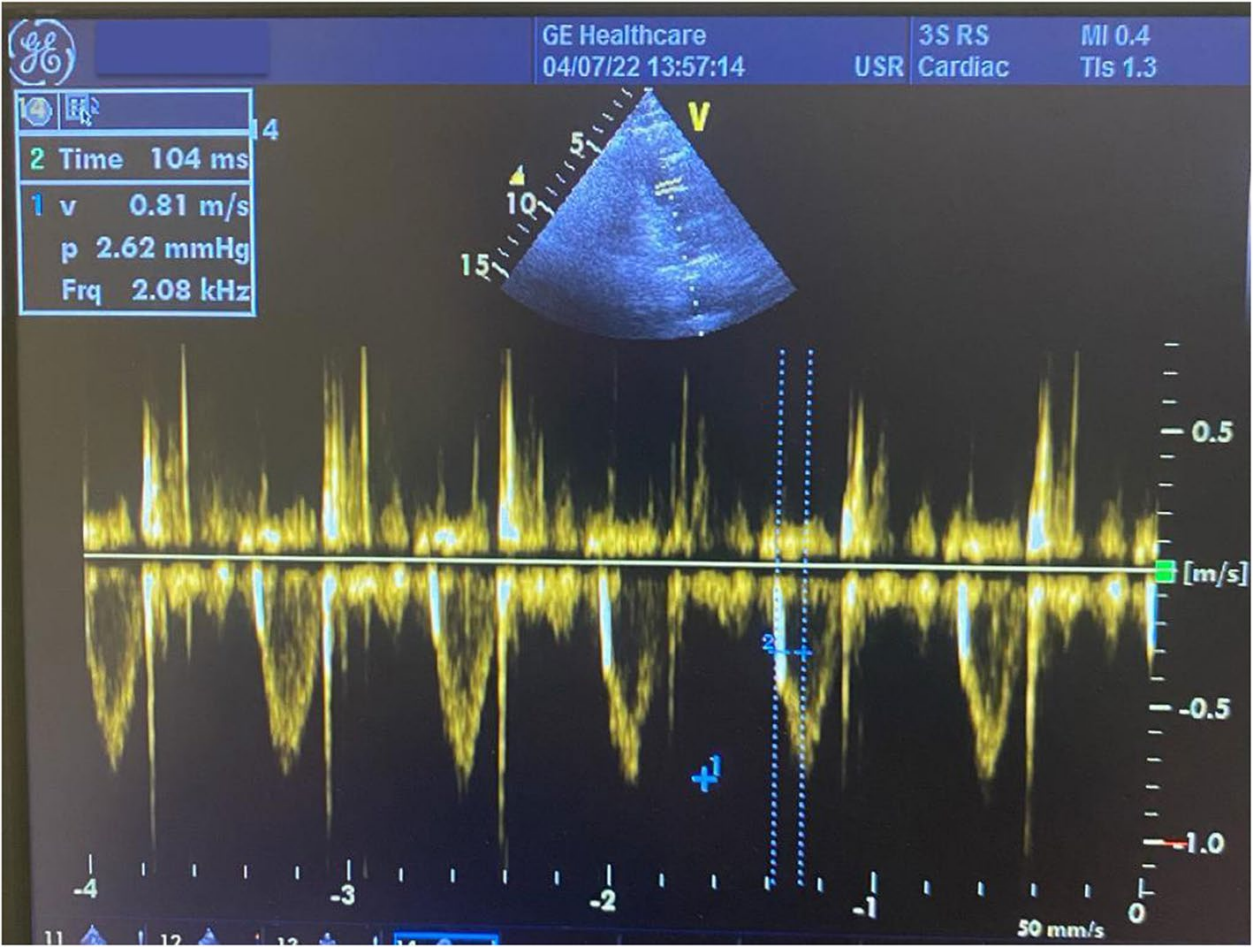
An example of measurement of pulmonary arterial stiffness

To evaluate PAS, a pulse Doppler recording was taken from the pulmonary artery flow through the parasternal short-axis view. For each measure, the average of at least three successive Doppler flow traces was assessed in order to counteract the possible impacts of breathing and the cardiac cycle on observations. From these recordings, the PAS was calculated with following formula: (maximum frequency shift of pulmonary flow) / (pulmonary flow acceleration time) (Fig. 1). The intraobserver and interobserver variability for PAS were detected as 3.2% and 3.6%, respectively.

### Laboratory analysis

All blood tests were performed after an 8- to 12-h overnight fast, biochemical markers and lipid profiles were measured. Standard analyzers were used to determine the results of hematological and biochemical parameters, respectively.

### Statistical analysis

All statistical analyses were carried out using R ver. 3.6.3 (R Foundation for Statistical Computing). The Kolmogorov–Smirnov test was used to determine normality. The mean ± standard deviation was used to represent variables with normal distribution, and median was used to represent variables with non-normal distribution (interquartile range). For continuous variables, the independent sample t-test or Mann–Whitney U-test was used, depending on the situation. Comparisons of categorical variables were made using the chi-square or Fisher exact test. A multivariable logistic analysis was carried out with covariates that were statistically meaningful (*P* < 0.05) in the univariable logistic regression analysis. Variance inflation factor and tolerance factor were used to test for multicollinearity, with variance inflation factor > 3 and tolerance value < 0.1 recognized as evidence of multicollinearity. The receiver operating characteristic curve was utilized to determine the capacity of PAS to distinguish patients with COVID-19 from the control group. With the Youden index, an effective cutoff value of PAS for discriminating ability was established.

## Results

In all, 201 post–COVID-19 patients and 195 healthy subjects were enrolled in this research. Table 1 presents the demographic information and laboratory results of the participants with and without COVID-19 history. Clinical features did not significantly differ between patients and controls. Patients with a prior history of COVID-19 infection had higher white blood cell (WBC) counts, neutrophil counts, and red cell distribution width compared with healthy subjects. Additionally, these patients had lower lymphocyte levels. We did not note any significant differences regarding other laboratory data.

**Table 1:** Demographic and laboratory features of patients (*n* = 306)

**Variable**	Control (*n* = 195)	COVID-19 (*n* = 201)	*P*-value
Age (yr)	49.2 ± 14.9	48.2 ± 16.3	0.520
Male sex	104 (53.3)	89 (44.3)	0.089
Body mass index (kg/m^2^)	27.6 ± 4.46	28.4 ± 4.36	0.075
Systolic blood pressure (mmHg)	112 ± 17.7	114 ± 14.0	0.199
Diastolic blood pressure (mmHg)	71.9 ± 8.99	72.6 ± 7.80	0.422
Heart rate (beats/min)	78.0 (72.0–86.0)	79.0 (70.0–86.0)	0.700
**Laboratory data**			
White blood cell counts (× 10^3^/μL)	7.80 (6.68–9.23)	8.10 (6.90–9.84)	0.045
Hemoglobin (g/dL)	14.0 (13.2–14.7)	14.1 (13.0–15.1)	0.162
Platelets (× 10^3^/μL)	257 ± 64.0	255 ± 80.0	0.751
Lymphocyte (× 10^**3**^/μL)	2.31 (1.88–2.80)	2.02 (1.28–2.71)	0.001
Neutrophil (× 10^**3**^/μL)	4.79 (3.82–5.97)	5.02 (4.06–7.01)	0.014
Monocyte (× 10^**3**^/μL)	0.43 (0.35–0.53)	0.46 (0.34–0.56)	0.460
**Red cell distribution width** (fL)	42.2 (40.1–44.6)	42.9 (40.8–45.4)	0.026
**Mean platelet volume** (fL)	9.80 (9.10–10.5)	9.70 (8.90–10.5)	0.634
Glucose (mg/dL)	115 ± 28.4	119 ± 29.1	0.155
Creatinine (mg/dL)	0.73 (0.64–0.84)	0.74 (0.60–0.95)	0.985
**C-reactive protein** (mg/dL)	6.46 (3.38–13.5)	8.23 (3.27–16.0)	0.100
Uric acid (mg/dL)	5.09 (4.10–5.55)	5.09 (4.10–6.30)	0.207
Aspartate transferase (U/L)	17.7 (15.0–21.0)	18.0 (14.0–22.4)	0.808
Alanine aminotransferase (U/L)	16.0 (12.0–24.0)	17.0 (13.0–23.1)	0.336
Sodium (mmol/L)	139 (136–140)	139 (136–140)	0.449
Potassium (mmol/L)	4.20 (3.96–4.36)	4.25 (3.90–4.50)	0.140
Magnesium (mmol/L)	1.90 (1.90–2.04)	1.93 (1.80–2.10)	0.725
Calcium (mmol/L)	9.20 (8.80–9.58)	9.20 (8.80–9.50)	0.609
Albumin (mg/dL)	4.20 (3.44–4.58)	4.25 (3.67–4.60)	0.311
Triglyceride (mg/dL)	160 (108–195)	164 (126–203)	0.190
High-density lipoprotein cholesterol (mg/dL)	42.0 (39.0–51.0)	41.1 (36.0–48.0)	0.066
Low-density lipoprotein cholesterol (mg/dL)	100 (82.5–123)	104 (87.4–131)	0.139

Values are presented as mean ± standard deviation, number (%), or median (interquartile range)

Table 2 represent the detailed echocardiographic examinations of all participants. The PAS was found to be 8.56 ± 1.47 Hz/msec in the control group and 10.20 ± 4.11 Hz/msec in the post–COVID-19 group (*P* < 0.001). Moreover, pulmonary artery acceleration time was significantly lower in patients with a prior history of COVID-19. On the other hand, pulmonary velocity, LA diameter, A wave, and systolic PA pressure (sPAP) were significantly elevated among these patients.

**Table 2:** Echocardiographic properties of patients (*n* = 306)

**Variable**	Control (*n* = 195)	COVID-19 (*n* = 201)	*P*-value
Pulmonary velocity (m/sec)	891 ± 119	941 ± 188	0.002
Pulmonary acceleration time (msec)	106 ± 17.7	100 ± 25.8	0.005
Pulmonary artery stiffness (Hz/msec)	8.56 ± 1.47	10.20 ± 4.11	< 0.001
Left ventricle ejection fraction (%)	61.70 ± 1.40	61.40 ± 2.53	0.167
Left ventricle end-diastolic diameter (cm)	4.35 ± 0.52	4.44 ± 0.64	0.115
Left ventricle end-systolic diameter (cm)	2.88 ± 0.46	2.85 ± 0.57	0.504
Interventricular septum thickness (mm)	10.3 ± 1.90	10.1 ± 1.89	0.325
Posterior wall (mm)	9.29 ± 1.84	9.54 ± 5.22	0.535
Left atrium diameter (cm)	3.2 (2.9–3.5)	3.4 (3.0–3.7)	0.001
E wave (m/sec)	0.75 (0.59–0.92)	0.72 (0.58–0.88)	0.385
A wave (m/sec)	0.68 (0.56–0.79)	0.71 (0.60–0.87)	0.049
RV S wave (m/sec)	0.08 (0.07–0.10)	0.08 (0.07–0.10)	0.407
e’ (m/sec)	0.13 (0.10–0.15)	0.12 (0.08–0.15)	0.183
a’ (m/sec)	0.09 (0.07–0.11)	0.09 (0.07–0.11)	0.063
RV e’ (m/sec)	0.11 (0.08–0.14)	0.10 (0.08–0.12)	0.465
RV a’ (m/sec)	0.15 (0.12–0.18)	0.14 (0.12–0.18)	0.322
E/e’	6.09 (4.86–7.62)	6.12 (4.92–8.00)	0.477
Interventricular relaxation time (msec)	74.0 (63.0–89.0)	80.0 (66.0–99.0)	0.186
Deceleration time (msec)	182 (154–220)	191 (153–237)	0.163
RV MPI	0.56 (0.46–0.78)	0.56 (0.47–0.74)	0.929
sPAP (mmHg)	6 (5–14)	20 (17–27)	< 0.001
RVFAC (mm^2^)	34.5 (29.0–40.1)	34.3 (25.4–39.1)	0.064
RV basal diameter (cm)	2.8 (2.6–3.2)	3.0 (2.6–3.2)	0.540
RV longitudinal diameter (cm)	4.80 (4.55–5.45)	4.90 (4.60–5.50)	0.675
TAPSE (cm)	2.50 (2.20–2.60)	2.36 (2.16–2.56)	0.357

Values are presented as mean ± standard deviation or median (interquartile range)

*RV* Right ventricle, *MPI* Myocardial performance index, *sPAP* systolic pulmonary arterial pressure, *RVFAC* Right ventricle fractional area contraction, *TAPSE* Tricuspid annular plane systolic excursion

**Fig. 2:**
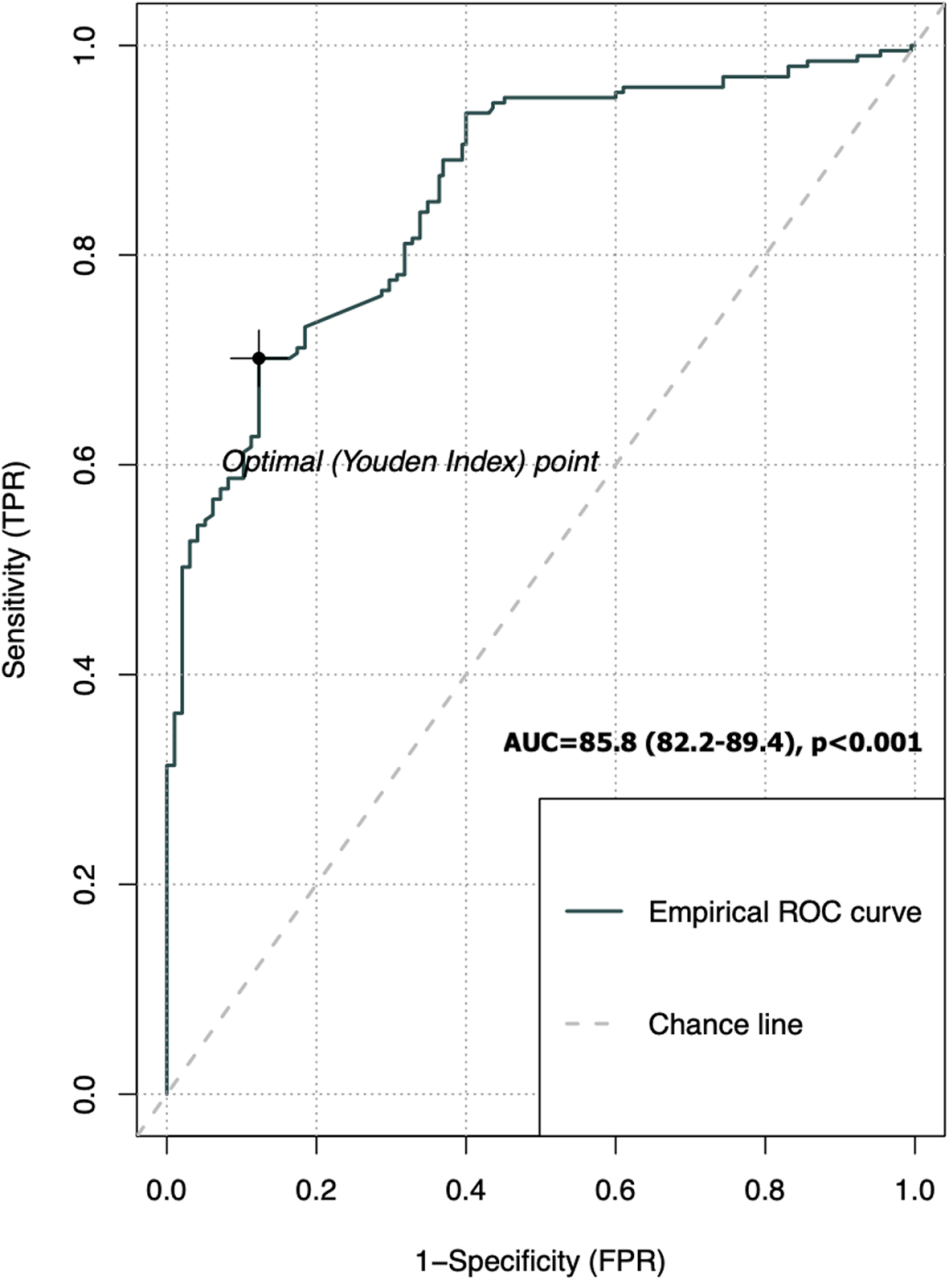
The ideal value of pulmonary arterial stiffness to detect a prior COVID-19 disease. ROC, receiver operating characteristic; AUC, area under the curve; TPR, true positive rate; FPR, false positive rate

By using univariable and multivariable logistic regression analysis, the independent impacts of likely echocardiographic characteristics linked to prior COVID-19 illness were assessed. Following the application of the univariable analysis, WBC counts, lymphocyte, neutrophil, red cell distribution width, sPAP, PAS, LA diameter, and RV fractional area contraction were associated with a prior COVID-19 illness. All abovementioned variables except WBC were included in the multivariable logistic regression analysis. Due to the presence of multicollinearity, WBC could not be added to the multivariable model. The multivariable analysis revealed that only PAS (odds ratio, 1.267; 95% confidence interval, 1.142–1.434; *P* < 0.001) and sPAP were independently linked with a previous COVID-19 disease (Table 3). LA was found to be different between the two groups; however, in multivariable analysis, it was not an independent parameter of prior COVID-19 infection. Receiver operating characteristic curve analysis showed that PAS had a higher discriminative ability between prior COVID-19 patients and control groups (area under the curve, 85.8; 95% confidence interval, 82.2–89.4; *P* < 0.001) (Fig. 2). The optimal cutoff value for detecting a prior COVID-19 disease for PAS was 10.1 (sensitivity, 70.2%; specificity, 87.7%).

**Table 3:** Logistic regression analysis for detecting independent association of variables with COVID-19 disease

**Variable**	Univariable			Multivariable		
	OR	95% CI	*P*-value	OR	95% CI	*P*-value
Lymphocyte (× 10^3^/μL)	0.738	0.591–0.915	0.006	1.006	0.754–1.345	0.972
Neutrophil (× 10^3^/μL)	1.150	1.063–1.254	0.001	1.034	0.931–1.159	0.547
RDW (fL)	1.061	1.017–1.110	0.007	1.054	0.997–1.118	0.068
sPAP (mmHg)	1.184	1.148–1.225	< 0.001	1.189	1.148–1.235	< 0.001
PAS (Hz/msec)	1.257	1.150–1.387	< 0.001	1.267	1.142–1.434	< 0.001
LA diameter	2.158	1.422–3.326	< 0.001	1.073	0.610–1.896	0.806
RVFAC (mm^2^)	0.974	0.951–0.997	0.014	0.986	0.954–1.011	0.220

*OR* Odds ratio, *CI* Confidence interval, *RDW* Red cell distribution width, *sPAP* systolic pulmonary arterial pressure, *PAS* Pulmonary artery stiffness, *LA* Left atrial, *RVFAC* Right ventricle fractional area contraction

## Discussion

The findings of this investigation revealed that PAS was significantly elevated in mild post–COVID-19 patients, which may indicate early changes on pulmonary vascular bed in such patients. The stiffening of the pulmonary arteries is a crucial element in the pathogenesis of PAH. Histological evidence of PAS in PAH includes the activation and proliferation of all cellular subtypes in the three layers of the pulmonary artery wall, including endothelial cells of the tunica intima and vascular smooth muscle cells of the tunica media. The endothelial cell layer in the intima is active and physically and functionally associated with the media and adventitia's fibroblasts and myofibroblasts, resulting in the production and stromal deposition of extracellular media components. Both myofibroblasts from the adventitia and activated smooth muscle cells may migrate into the tunica media's endothelial cell layer. This complex interaction between the cells and the extracellular media elements results in an intimal concentric hyperplasia of the tunica. This modification of the pulmonary vasculature leads to a progressive decrease in arterial compliance and the development of vascular stiffness [12].

Although right heart catheterization is the preferred technique for diagnosing, evaluation of PAS with noninvasive techniques including echocardiography, computed tomography, and cardiac magnetic resonance imaging have been developed in consideration of the expense and potential risks of right heart catheterization [13, 14]. The RV function has been associated to PAS, and this relationship may be used to estimate functional capacity in PAH. Studies show that the PAS is elevated in the early stages of PAH, and it may be used to detect the disease early. Öz et al. [15] showed that PAS might be helpful in the early detection of abnormalities in the pulmonary vascular bed even while RV function or sPAP levels were normal in cirrhotic patients. Another study found that PAS values increased in polycystic ovarian syndrome patients, and it was associated with high homeostatic model assessment for insulin resistance (HOMA-IR) levels [16]. In the same study, it was reported that the same patient group had reduced RV-PA coupling, which was an indication of PA compliance that played a significant role in the etiology of PAH. Baysal and Has [17] revealed that PAS was higher in early stage asthmatic patients with subclinical RV dysfunction compared with controls. Similarly, Ozkececi et al. [18] discovered that obstructive sleep apnea syndrome patients without PAH had higher PAS levels. As far as we know, PAS was evaluated for the first time in the literature by echocardiographic method in patients who survived COVID-19 without hospitalization. This study may be the first to show that participants who survived COVID-19 without hospitalization had significantly higher PAS values compared to participants who had never had COVID-19.

Our investigation's results have clinical relevance. It is critical to identify pulmonary vascular changes as soon as possible that might signify clinically relevant RV dysfunction [19]. Clinicians have mostly focused on pulmonary artery pressure in the clinical management of patients, but once pulmonary artery pressure rises, it may be too late for optimal treatment. Early PAH diagnosis facilitates treatment and improves survival chances. We noticed that none of the subjects in this study had a definite PAH. However, the COVID-19 group's PAS levels were greater. We believe that PAS may identify early involvement in the pulmonary vasculature system in patients who survived COVID-19 since these individuals had higher PAS levels without RV functional impairment and an increase in sPAP.

Our research has a number of limitations. First, it was a regional study with a small number of participants. Another limitation of the study was that although the study population comprised outpatients with COVID-19 disease, it was not known whether the pulmonary involvement was present or not. In addition, it would be more valuable if we could conduct our study in the same patient group before and after COVID-19. However, since the PAS is not routinely evaluated in our clinic, we preferred healthy volunteers who had not been diagnosed with COVID-19 before and who had no COVID-19 symptoms in the last 6 months as the control group. Further long-term, larger-group and multicenter studies are needed to verify our results.

## Conclusions

This study showed that PAS values were elevated in patients with a history of COVID-19 in the post–COVID-19 period.

## Data Availability

Not applicable.

## Abbreviations

HOMA-IR: Homeostatic model assessment for insulin resistance
LA: Left atrial
MERS: Middle East respiratory syndrome
MPI: Myocardial performance index
PA: Pulmonary arterial
PAH: Pulmonary arterial hypertension
PAS: Pulmonary arterial stiffness
RV: Right ventricle
SARS: Severe acute respiratory syndrome
sPAP: Systolic pulmonary arterial pressure
TVCOt: Time from tricuspid valve closure to opening
WBC: White blood cell
